# Effectiveness of a flipped classroom combined with case-based learning in the clinical training of pediatric interns: a randomized controlled study

**DOI:** 10.3389/fmed.2026.1789024

**Published:** 2026-03-16

**Authors:** Zhiyan Hao, Ying Huang, Shan Cao, Yuqing Zhang, Dan Wang

**Affiliations:** 1 Department of Pediatrics, Shanghai Pudong Hospital (Pudong Hospital Affiliated to Fudan University), Shanghai, China; 2 Department of Urology, Shanghai Pudong Hospital (Pudong Hospital Affiliated to Fudan University), Shanghai, China

**Keywords:** case-based learning, clinical competence, flipped classroom, medical students, pediatric clinical training

## Abstract

**Background:**

To explore the effectiveness of a flipped classroom (FC) combined with case-based learning (CBL) in pediatric clinical training.

**Methods:**

115 undergraduate medical students undergoing pediatric clinical rotation were randomly assigned to either an intervention group receiving FC combined with CBL (FC + CBL) or a control group receiving traditional lecture-based teaching. Learning outcomes were objectively assessed using a theoretical knowledge test, Objective Structured Clinical Examination (OSCE), and routine performance evaluation. Subjective evaluation was conducted using the Self-Assessment Scale for Active Learning and Critical Thinking (SSACT) and a course satisfaction questionnaire. Correlation analysis was performed to explore underlying learning mechanisms.

**Results:**

Baseline characteristics were comparable between the two groups. In terms of objective assessment, the intervention group scored significantly higher than the control group in total score, theoretical knowledge, and OSCE (*p* < 0.05), while no significant difference was observed in routine performance. Regarding subjective assessment, the intervention group reported significantly higher scores than the control group in active learning, critical thinking, learning efficiency, teamwork and communication, problem-solving ability, practical skills training, learning motivation, and overall teaching effectiveness (*p* < 0.01). Correlation analysis revealed stronger positive associations between active learning, critical thinking, learning motivation, and objective performance outcomes in the intervention group compared to the control group.

**Conclusion:**

The FC + CBL approach significantly improves both objective learning outcomes and subjective learning experiences in pediatric clinical training. The enhancement of active learning and critical thinking abilities may play a key mediating role in this process.

## Introduction

Clinical clerkship is a pivotal stage during which medical students transition toward the professional role of a physician, and it also represents a critical phase in pediatric medical education (1). Unlike adult medicine, pediatric patients often have limited ability to accurately articulate subjective symptoms, disease progression tends to be more rapid, and clinical management relies heavily on holistic interpretation of the clinical context (2). These characteristics place higher demands on medical students’ clinical reasoning, self-directed learning, and comprehensive clinical competence (3). Conventional clinical teaching often resembles an apprenticeship model in which teachers primarily transmit experiential knowledge and textbook content, while students passively receive information; such an approach may fail to effectively integrate theoretical learning with clinical practice (4–6). As a consequence, learners may focus narrowly on performing procedures while overlooking differential diagnosis and clinical decision-making, which is unfavorable for developing broader clinical thinking and comprehensive clinical skills.

With the rapid advancement of science and technology, medical education has increasingly entered the era of digital and internet-enabled learning (7, 8) and the flipped classroom (FC) is precisely a teaching model that can adapt to these changes (9). In this approach, instructors curate and deliver pre-class learning materials using information technology and guide students to engage in active learning, literature searching, and reflective thinking. Classroom time is then restructured to shift learning ownership from the teacher to students, transforming traditional didactic lectures into interactive discussion-based learning, which may enhance higher-order thinking and learning engagement (9, 10). Case-based learning (CBL), which draws on the educational tradition of the “case method,” has been widely adopted across health professions education. CBL typically uses representative clinical cases to make tacit knowledge explicit and to promote analysis within specific clinical contexts, thereby strengthening independent thinking and problem-solving and potentially improving learning efficiency (11–13).

Although FC and CBL have demonstrated educational benefits individually, evidence regarding their combined application in pediatric clinical training remains limited. In particular, it is unclear whether an integrated FC + CBL approach can simultaneously improve objective learning outcomes—such as theoretical knowledge and clinical competence—and subjective outcomes, including active learning, critical thinking, and learner satisfaction. Furthermore, the mechanisms through which active learning processes might mediate these outcomes have yet to be systematically explored.

To address the limited evidence regarding the combined application of FC and CBL in pediatric clinical training—particularly its impact on both objective performance and learner-centered outcomes—we designed a randomized controlled trial in which undergraduate pediatric interns were allocated to either an integrated FC + CBL approach or traditional lecture-based teaching. This study aimed to evaluate the effects of the combined model on theoretical knowledge, clinical competence, and learning experiences, and to examine the relationships between active learning, critical thinking, and performance outcomes.

## Methods

### Study design

This study was a randomized controlled trial (RCT) conducted to evaluate the effectiveness of a FC + CBL in pediatric clinical training. Ethical approval was obtained from the Ethics Committee of Shanghai Pudong Hospital (Approval number: SPH-2023-089), and written informed consent was obtained from all participants. The study was conducted in accordance with the Declaration of Helsinki. To ensure that participation did not influence students’ official academic evaluation, all training and assessment activities conducted as part of this study were separate from their formal grading. Furthermore, to promote educational equity, students in the control group were provided the opportunity to access the FC + CBL teaching materials and sessions upon request after the study’s completion.

### Participants

Undergraduate interns who underwent pediatric internships at Pudong Hospital, Fudan University, between June 2023 and June 2024 for a duration of at least 6 weeks were enrolled in this study. Eligible participants included those who had participated in clinical internship or pre-internship training at the institution, were undertaking their first clinical rotation in pediatrics, and had an attendance rate exceeding 90% during the training period. Participants were excluded if they refused to participate, had absenteeism of 10% or higher, or withdrew for other reasons. Prior to group allocation, all participants completed a baseline assessment consisting of a pediatric knowledge test and a clinical case analysis examination to evaluate baseline knowledge and clinical reasoning ability.

The sample size was calculated prior to study initiation using G*Power software (version 3.1). Based on previous educational intervention studies evaluating flipped classroom approaches (14), a medium effect size (Cohen’s *d* = 0.5) was assumed for differences in objective learning outcomes between the two groups. With a two-tailed significance level of 0.05 and a statistical power of 80%, the minimum required sample size was estimated to be 51 participants per group. Considering a potential dropout rate of approximately 10%, the target enrollment was set at no fewer than 112 participants. Ultimately, 115 students were included in the study, which satisfied the calculated requirement.

### Teaching interventions

Students in the intervention group participated in course modules that combined flipped classroom and case-based learning. Each teaching case was divided into several clinical scenarios and distributed to students 1 week before class. Students were required to summarize patients’ symptoms and physical signs, propose potential diagnoses, explore underlying pathophysiological mechanisms, and formulate relevant clinical questions. Question generation was regarded as a core component of the learning process. During group discussions, basic questions were addressed through real-time online searches, whereas more complex or in-depth questions were assigned to individual students for further exploration. Students engaged in self-directed learning activities, including textbook reading, literature review, and participation in online courses, to acquire relevant knowledge and enhance independent learning skills. Within each group, the group leader collected and synthesized all questions raised by group members. Four to five students prepared a PowerPoint presentation covering selected questions and key learning points. During the in-class session, each group presented its findings, followed by group discussion. The instructor subsequently conducted an in-depth case analysis and provided comprehensive feedback, with particular emphasis on students’ strengths, originality, and innovative thinking. The curriculum focused on five core pediatric infectious diseases: hand-foot-and-mouth disease, rotavirus infection, community-acquired pneumonia, measles, and varicella. Teaching content emphasized case management, physical examination, diagnostic reasoning, and pediatric clinical procedures. Standardized teaching materials were provided, and all sessions were delivered by the same instructor to ensure instructional consistency and minimize inter-teacher variability.

Students in the control group received traditional lecture-based teaching (TBL). Instructors delivered theoretical instruction using multimedia presentations and demonstrated clinical procedures through case illustrations. Students practiced procedures under instructor supervision and completed routine assignments. Instructors assessed students’ understanding through targeted questioning and provided immediate feedback. Teaching focused on etiology, clinical manifestations, treatment strategies, and precautions related to pediatric infectious diseases. Students analyzed clinical conditions, summarized key points, and completed written case reports under instructor guidance. Table 1 summarizes the key similarities and differences between the FC + CBL and traditional lecture-based approaches.

**Table 1:** Comparison of teaching strategies between FC + CBL and traditional lecture-based groups.

Component	Intervention group	Control group
Pre-class preparation	Students received clinical cases in advance, summarized symptoms, proposed diagnoses, explored pathophysiology, and generated questions	Preparation limited to assigned readings
Self-directed learning	Independent study via textbooks, literature, and online resources; group leaders synthesized questions for presentations	Routine homework and guided reading
Group discussion & collaboration	Small-group discussions addressing questions; complex questions assigned individually; peer feedback	Limited in-class Q&A; mainly instructor-led
In-class session	Group presentations followed by instructor-led in-depth case analysis and feedback	Instructor lectures with procedural demonstration; supervised practice
Content focus	Five core pediatric infectious diseases; emphasis on case management, physical exam, diagnostic reasoning, clinical procedures	Same diseases; focus on etiology, clinical manifestations, treatment, and precautions
Instructor role	Facilitator and feedback provider, guiding reflection and discussion	Knowledge transmitter; demonstrates procedures and answers questions
Assessment alignment	Pre-class and in-class activities linked to theoretical knowledge and OSCE; promotes active learning and critical thinking	Standard curriculum; practice and instructor guidance
Follow-up opportunities	Pre-class materials reusable for review and reinforcement	Standard review

### Outcome measures

#### Objective assessment components

All students completed post-course assessments consisting of three components: theoretical knowledge, clinical competence, and routine performance. The total score was 100 points, with theoretical knowledge and clinical competence each accounting for 40% of the total score, and routine performance accounting for 20%.

Theoretical knowledge was assessed using a written examination comprising multiple-choice questions, multiple-answer questions, true/false items, and short-answer questions. The exam questions were developed by the pediatric teaching team based on standard textbooks and clinical guidelines, and were reviewed by three senior pediatric educators to ensure content validity. A pilot test on 10 students confirmed clarity and relevance, and the internal consistency of the test in this study was Cronbach’s *α* = 0.82.

Clinical competence was assessed using an Objective Structured Clinical Examination (OSCE) based on pediatric infectious disease cases (15). The OSCE comprised five structured stations (20 points each) assessing medical history taking, physical examination, clinical reasoning, documentation, and clinical decision-making. Standardized scoring checklists were developed and reviewed by pediatric faculty to ensure content validity. Examiners underwent standardized training, and inter-rater reliability in this study was acceptable (intraclass correlation coefficient [ICC] = 0.81).

Routine performance was evaluated by instructors based on attendance, participation, responsiveness, quality of case documentation, and engagement in clinical activities, with a maximum score of 100 points. The criteria were adapted from the institution’s standard clerkship assessment guidelines and pilot-tested to ensure clarity.

#### Subjective assessment components

Subjective assessment was conducted using the Self-Assessment of Self-Directed Learning and Critical Thinking (SSACT) scale to assess students’ active learning and critical thinking (16). The SSACT was translated into Chinese following standard forward-backward translation procedures, reviewed by three educational experts for content validity, and pilot-tested in 10 students to ensure cultural appropriateness. In the current study, Cronbach’s *α* was 0.87 for the Active Learning domain and 0.84 for the Critical Thinking domain.

A course satisfaction questionnaire comprising eight items was used to evaluate students’ perceptions of theoretical knowledge understanding, learning efficiency, overall teaching effectiveness, teamwork and communication skills, problem-solving ability, practical skills training, motivation, and overall satisfaction. Each item was rated on a five-point Likert scale ranging from 1 (very dissatisfied) to 5 (very satisfied), with overall satisfaction defined as the proportion of students rating the teaching method as “satisfied” or “very satisfied.” This questionnaire was developed by the research team based on prior literature and expert consultation, and pilot-tested in 10 students to confirm clarity and relevance. The internal consistency of the questionnaire in this study was Cronbach’s *α* = 0.87. All questionnaires were completed anonymously via an online survey platform.

### Statistical analysis

**Figure 1:**
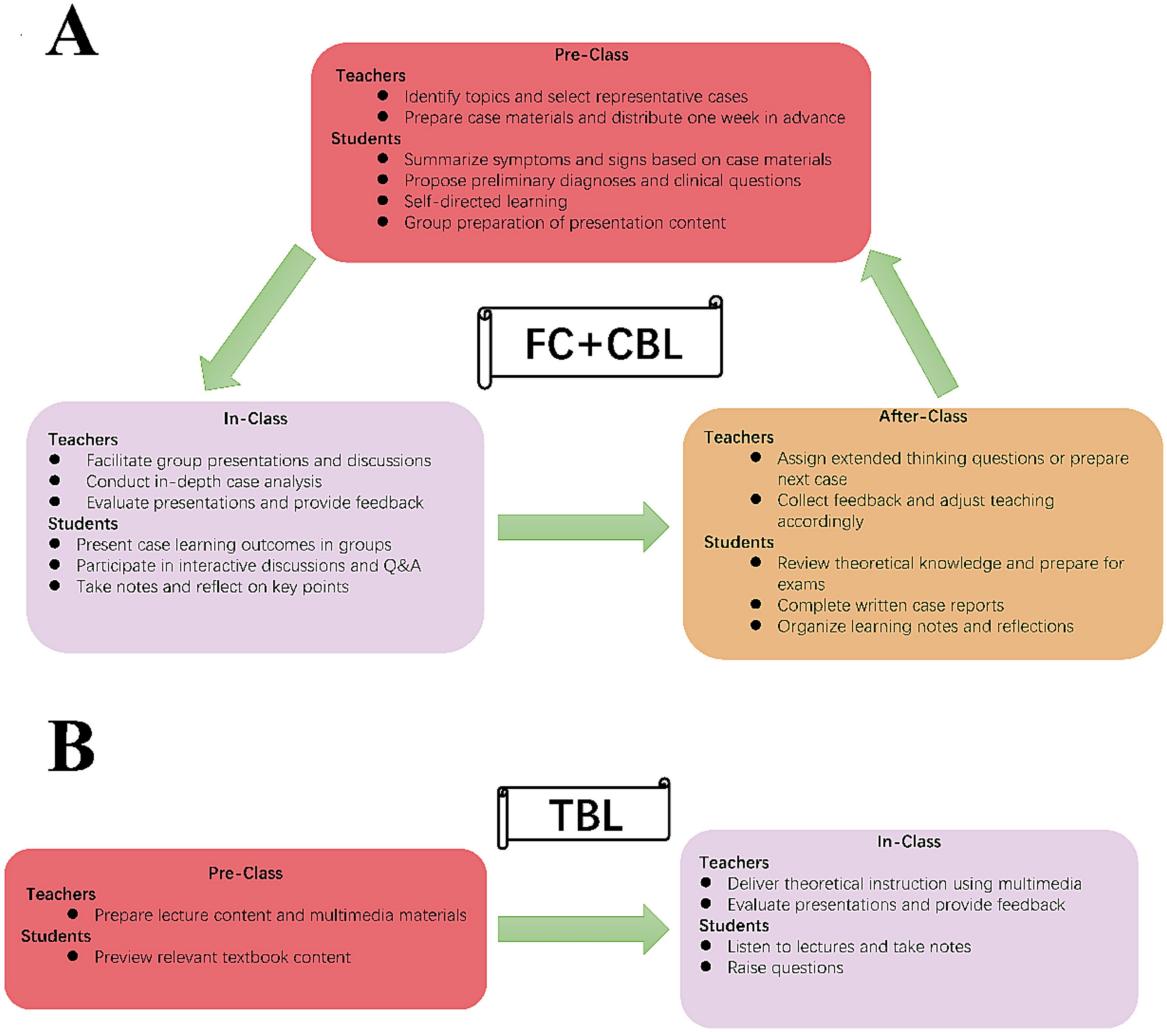
The schematic diagram. **(A)** Intervention group; **(B)** control group.

All statistical analyses were performed using SPSS version 26.0 (IBM Corp., Armonk, NY, USA) and R software (version 4.5.2; R Foundation for Statistical Computing). Continuous data with a normal distribution are presented as mean ± standard deviation (SD) and were compared using independent samples t-tests. Categorical data are presented as frequencies and were analyzed using the chi-square (*χ*^2^) test. Subjective assessment data (Likert-scale items) were analyzed using the non-parametric Mann–Whitney U test. To evaluate the inter-relationships among variables, Pearson’s correlation analysis was conducted separately for each group. A two-tailed *p*-value < 0.05 was considered statistically significant (Figure 1).

## Results

### Baseline characteristics

A total of 54 students were allocated to the intervention group and 61 to the control group. Participants were aged between 20 and 22 years. As shown in Table 2, no significant differences were observed between groups in age (*Z* = −0.412, *p* = 0.684), sex distribution (*χ^2^* = 0.033, *p* = 0.861), baseline pediatric knowledge scores (*t* = −0.925, *p* = 0.367), or clinical case analysis scores (*t* = 0.603, *p* = 0.552), indicating comparable baseline characteristics.

**Table 2:** Baseline characteristics of participants.

Characteristic	Intervention group (*n* = 54)	Control group (*n* = 61)	*Z/χ^2^*/*t*	*p*
Age, years	21 (20, 22)	21 (20, 22)	−0.412	0.684
Sex (female/male)	29/25	34/27	0.033	0.861
Pediatric knowledge test score	48.72 ± 5.76	49.68 ± 4.86	−0.925	0.367
Clinical case analysis score	74.53 ± 6.16	73.81 ± 6.88	0.603	0.552

### Objective assessment outcomes

**Figure 2:**
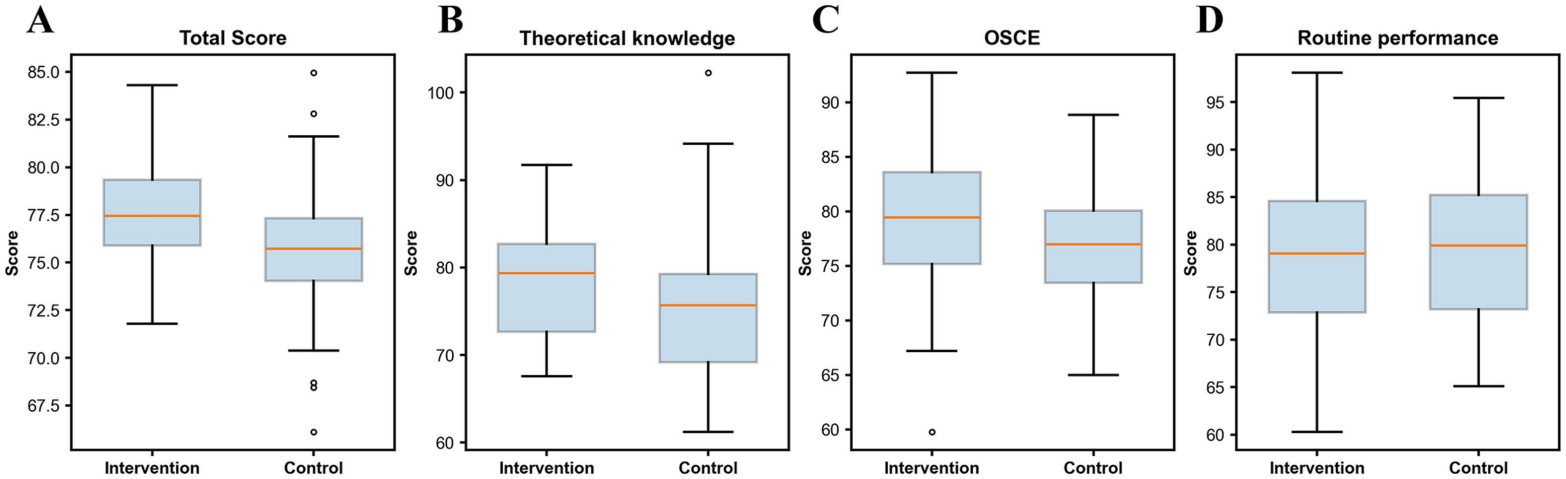
Distribution of objective assessment scores by teaching method. Box plots comparing the distribution of total scores **(A)**, theoretical knowledge scores **(B)**, OSCE scores **(C)**, and routine performance scores **(D)** between the intervention group and the control group.

**Figure 3:**
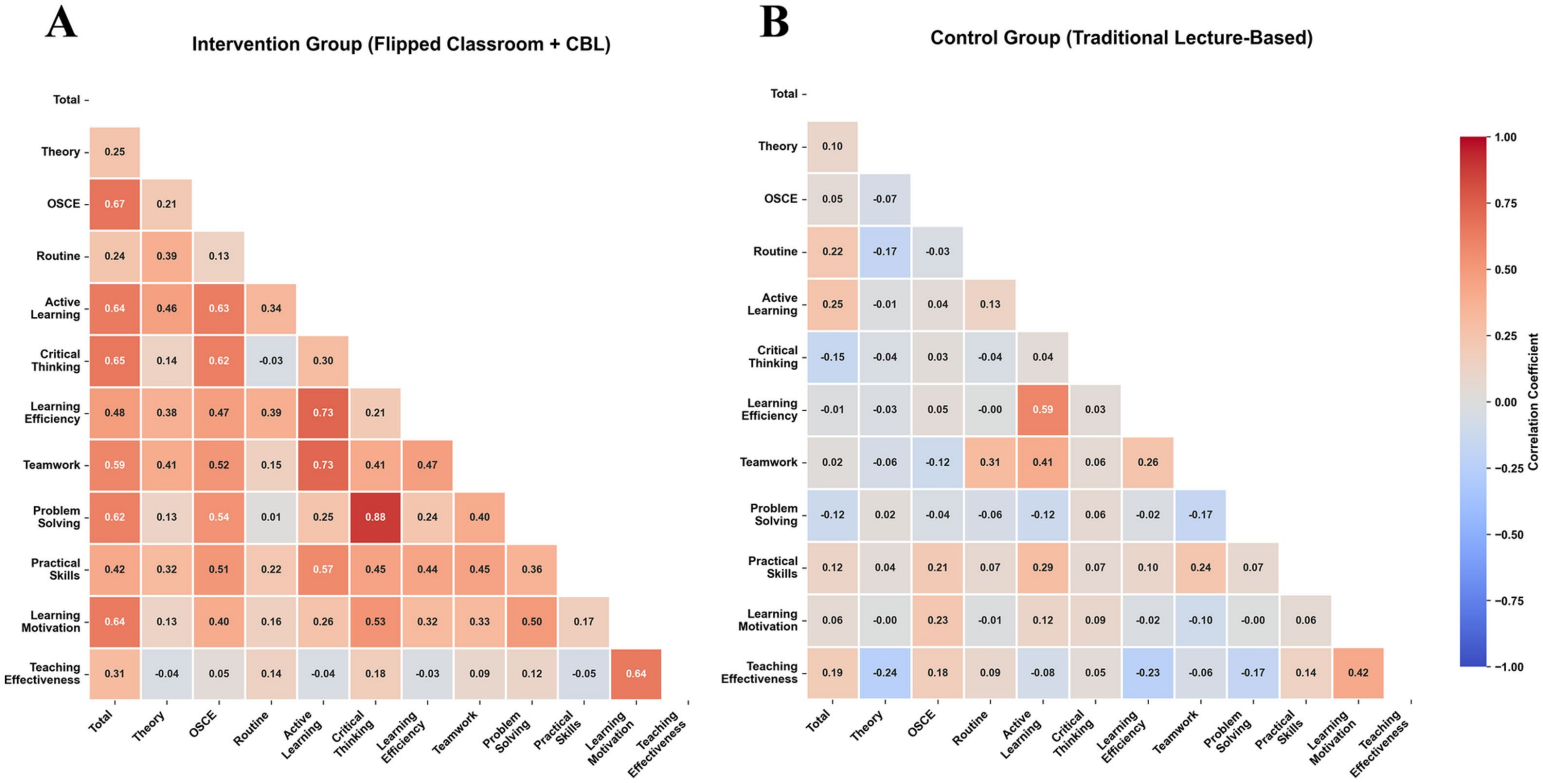
Comparison of correlation matrices. **(A)** Intervention group; **(B)** Control group.

The comparison of objective evaluation scores between the intervention group and the control group is shown in Table 3 and Figure 2. The intervention group achieved higher total scores (77.53 ± 2.99 vs. 75.88 ± 3.49, *t* = 2.17, *p* = 0.007), theoretical knowledge scores (78.23 ± 6.14 vs. 75.33 ± 7.42, *t* = 2.26, *p* = 0.025), and OSCE scores (79.34 ± 6.64 vs. 77.10 ± 4.58, *t* = 2.02, *p* = 0.045) compared with the control group. Routine performance scores did not differ significantly (79.60 ± 8.68 vs. 79.71 ± 7.54, *t* = −0.07, *p* = 0.946) (Figure 3).

**Table 3:** Comparison of objective assessment scores between the two groups.

Outcome	Intervention group (*n* = 54)	Control group (*n* = 61)	*t*	*p*
Total scores	77.53 ± 2.99	75.88 ± 3.49	2.17	0.007
Theoretical knowledge score	78.23 ± 6.14	75.33 ± 7.42	2.26	0.025
OSCE score	79.34 ± 6.64	77.10 ± 4.58	2.02	0.045
Routine performance score	79.60 ± 8.68	79.71 ± 7.54	−0.07	0.946

### Subjective assessment outcomes

Analysis of SSACT scores revealed significantly higher active learning (4.66 ± 0.69 vs. 3.44 ± 0.52, *t* = 9.98, *p* < 0.001) and critical thinking (4.12 ± 0.57 vs. 3.17 ± 0.45, *t* = 9.56, *p* < 0.001) in the intervention group (Table 4). Similarly, the course satisfaction questionnaire showed significantly higher ratings for learning efficiency (*t* = 2.80, *p* = 0.006), teamwork and communication (*t* = 2.91, *p* = 0.004), problem-solving (*t* = 3.16, *p* = 0.002), practical skills (*t* = 3.37, *p* = 0.001), learning motivation (*t* = 4.62, *p* < 0.001), and teaching effectiveness (*t* = 3.74, *p* < 0.001) in the intervention group (Table 5).

**Table 4:** Comparison of SSACT scores between the two groups.

SSACT domain	Intervention group (*n* = 54)	Control group (*n* = 61)	*t*	*p*
Active learning	4.66 ± 0.69	3.44 ± 0.52	9.98	<0.001
Critical thinking	4.12 ± 0.57	3.17 ± 0.45	9.56	<0.001

**Table 5:** Comparison of course satisfaction questionnaire scores.

Questionnaire item	Intervention group (*n* = 54)	Control group (*n* = 61)	*t*	*p*
Learning efficiency	4.16 ± 0.53	3.89 ± 0.50	2.80	0.006
Teamwork communication	4.42 ± 0.62	4.07 ± 0.67	2.91	0.004
Problem solving	4.35 ± 0.62	3.88 ± 0.90	3.16	0.002
Practical skills	4.23 ± 0.59	3.79 ± 0.76	3.37	0.001
Learning motivation	4.74 ± 0.85	3.95 ± 0.97	4.62	<0.001
Teaching effectiveness	4.32 ± 0.78	3.75 ± 0.84	3.74	<0.001

### Correlation analysis outcomes

**Figure 4:**
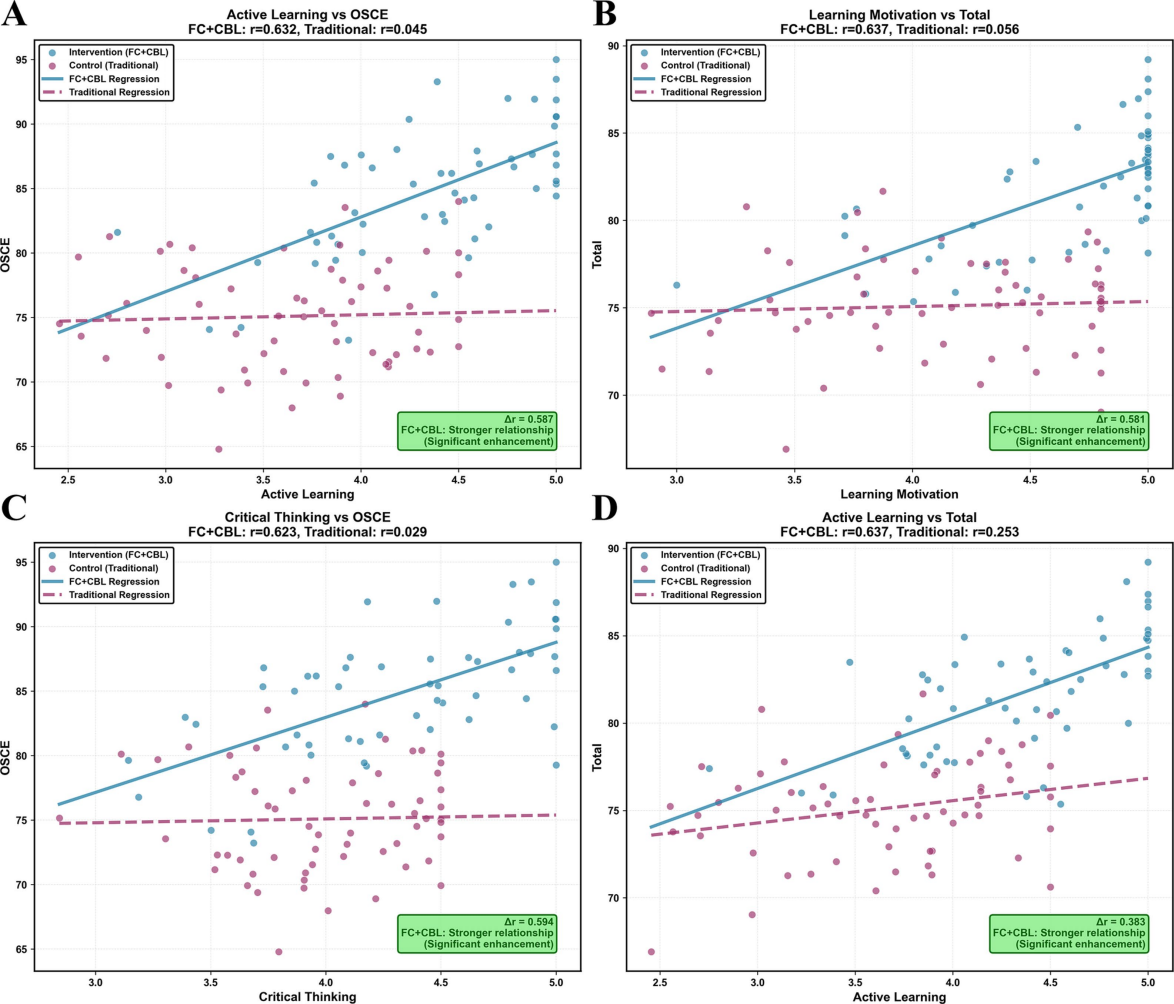
Key correlations between learning skills and performance. **(A)** Active learning vs. OSCE scores; **(B)** learning motivation vs. total score; **(C)** critical thinking vs. OSCE scores; **(D)** active learning vs. total score.

To explore the potential impact of teaching methods on learning mechanisms, correlation analyses were conducted separately for each group. In the intervention group, key learning skills—including active learning, learning motivation, and critical thinking—demonstrated moderate to strong positive correlations with objective assessment scores, particularly OSCE and total scores (*r* = 0.50–0.68, all *p* < 0.01). By contrast, correlations in the control group were weaker (*r* = 0.20–0.38, all *p* > 0.05). These differences were further illustrated in scatter plots (Figure 4), where the intervention group exhibited steeper regression slopes and tighter clustering of data points along the trend lines for core skill–outcome pairs, highlighting the stronger association between active learning processes and performance outcomes under the FC + CBL approach.

## Discussion

In this randomized controlled study, we evaluated the educational effects of an integrated FC + CBL model in pediatric clinical training. Objective learning outcomes, including theoretical knowledge, OSCE performance, and routine clinical tasks, were assessed without affecting students’ official academic grades, ensuring that evaluation could be conducted within the intervention context. The structured FC + CBL framework, incorporating pre-class preparation, active case discussions, and in-class group presentations, provided opportunities for deeper cognitive processing and skill acquisition. These findings align with prior studies reporting that flipped classroom interventions improve knowledge retention and engagement (17, 18), and that case-based learning enhances clinical reasoning and applied skills (19, 20). Unlike previous studies that typically evaluated either flipped classroom or CBL independently, our study examined the combined FC + CBL approach, demonstrating that integrating these methods may offer additive benefits by simultaneously fostering active preparation, collaborative problem-solving, and guided reflection.

The intervention also aimed to cultivate active learning, critical thinking, and learner satisfaction. Participants reflected systematically on clinical cases, generated and discussed questions in small groups, and collaboratively prepared presentations, creating a scaffolded learning environment emphasizing autonomy. Prior research has shown that small-group discussions and case-based inquiry improve motivation, analytical reasoning, and self-directed learning in medical education (21, 22). Our findings extend these observations by linking structured pre-class activities with in-class engagement, suggesting that FC + CBL provides a coherent framework for aligning student-centered learning with measurable subjective outcomes such as learning efficiency, teamwork, and perceived teaching effectiveness. Compared with studies focusing solely on traditional lecture-based curricula, our results indicate that the combined approach may better support learners in constructing knowledge and developing higher-order cognitive skills within authentic clinical contexts.

To explore potential mechanisms, we examined correlations between active learning, critical thinking, and objective performance. Strong positive associations in the intervention group suggest that FC + CBL may enhance performance by reinforcing self-directed learning behaviors and higher-order cognitive skills. This is consistent with literature demonstrating that engagement in case-based discussions promotes analysis of clinical information, differential diagnosis formulation, and decision justification (23, 24). Moreover, the flipped classroom component reallocates foundational knowledge acquisition to pre-class activities, freeing cognitive capacity during in-class sessions for integration, application, and peer discussion—a principle supported by cognitive load theory (25, 26). These observations provide plausible pathways through which FC + CBL contributes to both objective and subjective learning outcomes.

Overall, these findings support the educational value of combining flipped classroom and case-based learning in pediatric clinical clerkships. By structuring learning around pre-class preparation, collaborative inquiry, and guided case analysis, FC + CBL promotes knowledge acquisition while cultivating critical thinking and self-directed learning, essential competencies for clinical practice.

This study has several limitations. It was conducted at a single center with a modest sample size, limiting generalizability. Assessments were performed immediately after the intervention, so long-term retention and skill transfer were not evaluated. Although randomization was applied, potential influences from the Hawthorne effect or instructor expectancy bias cannot be entirely excluded. Finally, formal mediation analyses were not conducted; future research should employ multi-center designs and statistical mediation or structural equation modeling to clarify causal pathways and confirm the generalizability of these results.

## Conclusion

In conclusion, this randomized controlled study indicates that integrating a flipped classroom with case-based learning in pediatric clinical education is associated with significant improvements in theoretical knowledge, clinical competence, and learner-centered outcomes compared with traditional lecture-based teaching. The findings suggest that this instructional approach may enhance academic performance by promoting active engagement, self-directed learning, and critical thinking. Although further multicenter studies with larger samples and long-term follow-up are needed to confirm the durability and generalizability of these effects, the FC + CBL model represents a promising pedagogical strategy for optimizing undergraduate pediatric clinical training.

## Data Availability

The raw data supporting the conclusions of this article will be made available by the authors without undue reservation.
